# Evaluate the Structural and Physicochemical Properties of Exopolysaccharides Produced by *Bacillus halotolerans* Isolated from Locally Sourced Vegetables

**DOI:** 10.3390/polym16060759

**Published:** 2024-03-10

**Authors:** Yutian Dai, Min Xu, Zhijiang Zhou, Ye Han

**Affiliations:** School of Chemical Engineering & Technology, Tianjin University, Tianjin 300350, China; yutian_dai@163.com (Y.D.); zzj@tju.edu.cn (Z.Z.)

**Keywords:** characterization, *Bacillus subtilis*, exopolysaccharides

## Abstract

In this study, a *Bacillus halotolerans* (*B. halotolerans*) strain DT1 capable of producing exopolysaccharides (EPS) was isolated from dried cabbages of Tianjin, a local fermented vegetable product. Three distinct polysaccharide fractions were isolated from the fermentation broth of DT1, namely, DT1-0, DT1-2, and DT1-5. The structural composition and properties of these fractions were investigated. The predominant EPS, DT1-0, was identified as a novel heteropolysaccharide composed of fructose and glucose with branched structures. The repeating unit was determined to be [4)-α-D-Glcp-(1→6)-α-D-Glcp-(1→6)-β-D-Fruf-(2→6)-β-D-Fruf-(2→6-)-β-D-Fruf-(2→], with fructose and glucose connected by β-(2→1) and α-(1→4) glycosidic linkages between the third fructose and the first glucose, respectively. The molecular weight (*Mw*) was estimated to be 4.253 × 10^3^ Da. DT1-0 presented a smooth and porous surface structure as observed through SEM and exhibited a water-holding capacity of 504 ± 5.3%, maximum thermal stability at 245 °C, and an oil-holding capacity of 387 ± 1.9% for coconut oil. DT1-2 was identified as a fructooligosaccharide. DT1-5 was characterized as a polysaccharide composed of glucose and fructose. In conclusion, these findings provide substantial support for the further application of *B. subtilis* strain DT1 and its EPS fractions, DT1-0, DT1-2, and DT1-5, as potential alternatives for functional food additives or ingredients.

## 1. Introduction

The preserved dried cabbages of Tianjin are produced by slicing cabbages into fine shreds, followed by sun-drying and mixing with salt and minced garlic. The mixture is then packed into ceramic jars and sealed for a six-month fermentation period [1]. The production process of preserved dried cabbages is a mixed microbial natural fermentation. The principal microorganisms implicated in this process include *Lactic acid bacteria* (LAB) and *Bacillus* species. Among LAB, prominent genera include *Weissella*, *Leuconostoc*, and *Lactobacillus* [2], whereas *Bacillus* species encompass *Bacillus subtilis (B. subtilis*) and *Bacillus halotolerans (B. halotolerans)*. The *Bacillus*, characterized as Gram-positive bacteria, are commonly employed in the production of fermented foods such as natto and fermented soya beans with robust growth and reproductive capabilities. Certain *Bacillus* exhibit probiotic attributes and are approved for human consumption [3], and more importantly, a subset of *Bacillus* exhibits the ability to produce exopolysaccharides (EPS) [4]. The EPS produced by *Bacillus* demonstrates excellent physicochemical and biological properties, serving as biocoagulants, bioemulsifiers, heavy metal chelators, and antiviral agents [5]. As an example, EPS produced by halotolerant *B. subtilis LR-1*, consisting of glucose and mannose, exhibits excellent antioxidant prowess and thermal stability and demonstrates strong synergistic thickening properties when combined with kappa-carrageenan [6].

Under production conditions with a salt concentration of 16% for preserving dried cabbages, strains that play a role during the fermentation process often possess salt-tolerant characteristics. Studies have identified diverse microorganisms from preserved dried cabbages, including *Lactobacillus plantarum*, *Lactobacillus casei*, *Streptococcus lactis (S. lactis*), *Streptococcus cremoris*, and *Enterococcus durans*. Among these, *S. lactis* exhibited high salt tolerance. Hence, it can be inferred that *B. halotolerans* species are likely to be present in this high-salt fermentation system. Moreover, high-salt conditions hint at the presence of a salt fermentation ecosystem in preserved dried cabbages [7]; *Bacillus* species that thrive under extreme temperatures, pH, and salinity conditions tend to produce substances in order to adapt to such circumstances, such as extremozymes and EPS; and these microorganisms and their metabolic products collectively form the molecular mechanisms that facilitate adaptation to extreme physicochemical conditions. It is worth noting that distinct sources of *Bacillus* produce EPS with different structures, which may determine their divergent functionalities and application value. Hence, a comprehensive understanding of EPS structural composition and characteristics is pivotal in predicting specific application domains. 

However, research evaluating the structural and physicochemical properties of EPS produced by salt-adapted *Bacillus* in high-salinity environments remains limited. Because of its high salt production requirements, dried cabbages of Tianjin are very likely to cultivate one or more undiscovered native *B. halotolerans*. This study aims to isolate and identify *B. halotolerans* from locally sourced vegetables for the production of new EPS; a comprehensive assessment will be conducted on the structure and functionality of these strains. Furthermore, preliminary applications of EPS in food production will also be explored.

## 2. Method

### 2.1. Preserved Dried Cabbages of Tianjin

The preserved dried cabbages of Tianjin, China were procured and subsequently stored at −20 °C in the laboratory.

### 2.2. Screening for EPS-Producing Microorganisms

A certain volume of culture broth of preserved dried cabbages was diluted with physiological saline to concentrations ranging from 10^−4^ to 10^−7^. The diluted solutions were evenly spread onto LB-S agar medium (10 g/L tryptone, 5 g/L yeast extract, 10 g/L NaCl, 50 g/L sucrose, and 20 g/L agar) and incubated at 37 °C for 13 h. Colonies exhibiting mucoid characteristics were selected on LB-S culture broth with streaking inoculation and further cultured until a single mucoid-producing colony was obtained. The colony was preserved in a 30% (*v*/*v*) glycerol suspension and stored at −20 °C.

### 2.3. Identification of the Strain

The strain was subjected to morphological characterization and 16S rDNA sequencing. Colonial morphology was observed according to Bergey’s *Manual of Determinative Bacteriology*, and various tests were also conducted, including gram staining, catalase hydrolysis test, arginine double hydrolase test, gelatin liquefaction test, and starch hydrolysis test. Genomic DNA was extracted using a bacterial genome DNA extraction kit (Tiangen Biotech, Beijing, China), and the 16S rDNA gene was amplified using universal bacterial primers 16F: 5′-AGAGTTTGATCCTGGCTCAG-3′ and 16R: 5′-ACGGTTACCTTGTTACGACTT-3′. The PCR products were sequenced and then compared with reference sequences available in the NCBI database. Finally, a neighbor-joining phylogenetic tree based on genetic distance was constructed using the MEGA (version 7.0, Mega Limited, Auckland, New Zealand).

### 2.4. Isolation and Purification of EPS

The isolation and purification of EPS were conducted with slight modifications to the method established by Yuzhen Wang et al. in 2022 [8]. The strains were initially inoculated into 100 mL of LB culture broth and shaken during cultivation (37 °C, 180 rpm, 12 h). Subsequently, the seed liquid was inoculated into 2L LB medium containing 20% (*w*/*v*) sucrose for EPS production (37 °C, 180 rpm, 40 h). At the same time, blank control (LB medium containing 20% sucrose, adding the same volume of normal saline) was set up and the treatment was the same as the experimental group. The supernatant was collected by centrifugation (10,000× *g*, 10 min), followed by precipitation with three times the volume of pre-cooled 95% ethanol for 24 h at 4 °C. The precipitate was collected, dissolved in distilled water, and treated using the Savage method (chloroform/butanol, 4:1, *v*/*v*) to remove proteins, yielding a crude polysaccharide solution. The solution was subjected to DEAE-52 cellulose column chromatography, from which elution fractions were collected utilizing solutions containing 0 M, 0.2 M, and 0.5 M NaCl, and each elution fraction was subjected to dialysis against deionized water for 2 days. Following this, a purification step involving Sephadex G25 gel filtration chromatography was performed and dialyzed for 2 d, and then freeze-dried. Through this method, three purified EPS were successfully obtained.

### 2.5. Determination of the Relative Molecular Weight (MW) of EPS

The determination of relative *Mw* was conducted using a Waters 2695 system equipped with a refractive index (RI) detector (Optilab T-Rex, Wyatt Technology, Santa Barbara, CA, USA) and a detection column (Ultrahydrogel linear Column, 10 µm, 7.8 mm × 300 mm, 1K-7M, 1/pk). The powder of EPS was dissolved in a 0.1 mol/L NaNO_3_ solution at a concentration of 5 mg/mL and subsequently filtered through a 0.22 μm pore size filter. Subsequently, a 30 μL sample was injected into the HPSEC-MALLS-RI system and eluted with a constant flow rate of 0.5 mL/min using a 0.1 mol/L NaNO_3_ mobile phase for 30 min.

### 2.6. Monosaccharide Composition Analysis

Utilizing the Thermo ICS 5000+ ion chromatography system (ICS 5000+, Thermo Fisher Scientific, Waltham, MA, USA), monosaccharide composition analysis was conducted using an electrochemical detector. A suitable quantity of polysaccharide samples was weighed with clean chromatography vials. Subsequently, 1 mL of 2M TFA was added, and the mixture was heated at 121 °C for 2 h. After drying the sample under nitrogen gas, it was subjected to a cleaning process with 99.99% methanol, followed by repeated drying. The chromatographic data were processed using the Chromeleon (version 7.2, Thermo Fisher Scientific, Waltham, MA, USA).

### 2.7. Glycosidic Linkage Analysis

The derivatives were analyzed using GC-MS (7890A-5977B, Agilent Technologies Inc., Santa Clara, CA, USA) with an HP-5MS capillary column (30 m × 0.25 mm × 0.25 μm, Agilent J&W Scientific, Folsom, CA, USA), and the temperature program was as follows: an initial column temperature of 50 °C was maintained for 1 min, followed by a ramp of 50 °C/min to 130 °C, then a ramp of 3 °C/min to 230 °C with a 2 min hold. The resulting methylated product was subjected to NMR detection, such as 2.9 operation. 

### 2.8. Fourier Transform Infrared Spectroscopy (FTIR)

The characterization of EPS functional groups was conducted using the Fourier Transform Infrared Spectrometer (Nicolet iS20, Thermo Scientific, Waltham, MA, USA) through the KBr pellet method. Purified EPS was mixed with dry KBr powder in a ratio of approximately 1:100, and the resulting mixture was pressed into transparent thin pellets for scanning. The Fourier Transform Infrared Spectrometer was configured to operate in the wavenumber range of 400–4000 cm^−1^, with a resolution of 4 cm^−1^, and each scan was performed with 32 accumulations.

### 2.9. Nuclear Magnetic Resonance Spectroscopy (NMR)

The purified EPS (50 mg) was subjected to three cycles of D_2_O exchange and finally re-dissolved in 1 mL of D_2_O in the NMR tube. The measurements were conducted on a Bruker AVANCE III HD 500 NMR spectrometer (Billerica, MA, USA), including one-dimensional nuclear magnetic resonance spectra: 1H and 13C, as well as two-dimensional nuclear magnetic resonance spectra: 1H-1H correlation spectroscopy (COSY), 1H-1H total correlation spectroscopy (TOCSY), 1H-13C heteronuclear multiple bond correlation (HMBC), and 1H-13C heteronuclear single quantum correlation (HSQC).

### 2.10. Thermal Stability Assessment

The thermal stability was investigated using a TGA 8000 thermogravimetric analyzer (PerkinElmer, Shelton, CT, USA), and the purified EPS was placed in an Al_2_O_3_ crucible under a nitrogen atmosphere with ramping the temperature at a rate of 10 °C/min.

### 2.11. Scanning Electron Microscopy (SEM) Analysis

The dried purified EPS powder was gold-coated under high vacuum conditions, and the surface morphology of the EPS film was observed using a Field Emission Scanning Electron Microscope (FEI Apreo S, Brno, Czech Republic). Images were captured at various magnification levels.

### 2.12. Atomic Force Microscopy (AFM) Analysis

Further characterization analysis of EPS was performed using an Atomic Force Microscope (Bruker Dimension Icon, Cambridge, MA, USA). The purified EPS was dissolved in deionized water at a concentration of 1 mg/mL, and 5 μL of the solution was extracted and dropped onto the surface of a silicon wafer sample carrier, followed by air-drying at room temperature. The Atomic Force Microscope was operated in tapping mode, employing a commercially available tapping silicon (Si) cantilever tip for image acquisition.

### 2.13. Water Solubility Index (WSI) Determination

The dried EPS (100 mg) was dispersed in 1 mL of deionized water and stirred uniformly for 2 h at 25 °C to obtain a solution. After centrifugation at 12,000× *g* for 10 min, the supernatant was collected, freeze-dried, and weighed. The Water Solubility Index (*WSI*) was calculated using the following formula:

$$WSI(\%)=\frac{dry\;solid\;weight}{total\;dry\;sample\;weight}\times 100$$

### 2.14. Water-Holding Capacity (WHC) Determination

The dried EPS (80 mg) was dispersed in 1.5 mL of deionized water and stirred for 2 h at 25 °C to obtain a solution. After centrifugation at 14,000× *g* for 30 min, the precipitate was collected. The calculation formula for water-holding capacity (*WHC*) is as follows:

$$WHC(\%)=\frac{water\;bound\;weight}{total\;dry\;sample\;weight}\times 100$$

### 2.15. Oil-Holding Capacity (OHC) Determination

The dried EPS (80 mg) was dispersed in 1.5 mL of olive oil and coconut oil, followed by 30 min of gentle agitation with intermittent shaking every 10 min. The suspension was centrifuged at 3500× *g* for 10 min, and the precipitate was collected and weighed [9]. The calculation formula for oil-holding capacity (*OHC*) is as follows:

$$OHC(\%)=\frac{oil\;bound\;weight}{total\;dry\;sample\;weight}\times 100$$

## 3. Results and Discussion

### 3.1. Identification of the Selected Strains

**Figure 1:**
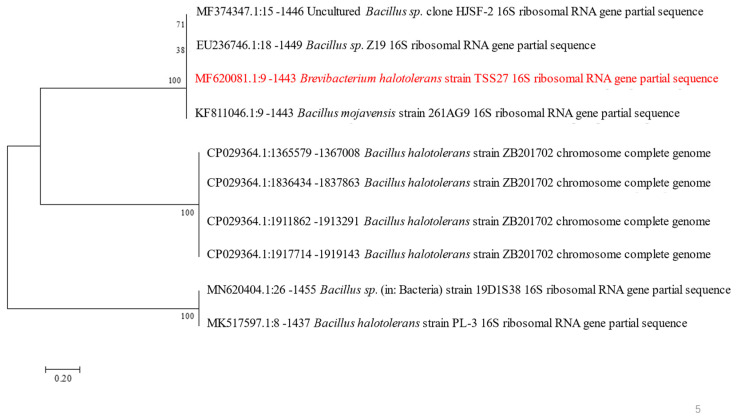
Phylogenetic tree of *B. halotolerans* strain DT-1.

The strain DT-1 isolated from preserved dried cabbages of Tianjin exhibited Gram-positive rod-shaped cells upon Gram staining. Partial physiological and biochemical tests were conducted, which indicated negative results for the oxidase test, negative results for the urease test, positive results for the gelatin liquefaction test, and positive results for the starch hydrolysis test. The strain exhibited growth within a range of 0% to 12% NaCl concentration, with the optimal concentration being 8%, and the highest similarity (96%) in terms of its 16S rDNA gene was observed with the sequence of *B. halotolerans* MF620081.1. The phylogenetic tree (Figure 1) depicts the closest relationship of this strain to *B. halotolerans* MF620081.1, suggesting its placement within the *Bacillus*.

### 3.2. Purification and Chemical Composition of Polysaccharides DT1-0, DT1-2, and DT1-5

**Figure 2:**
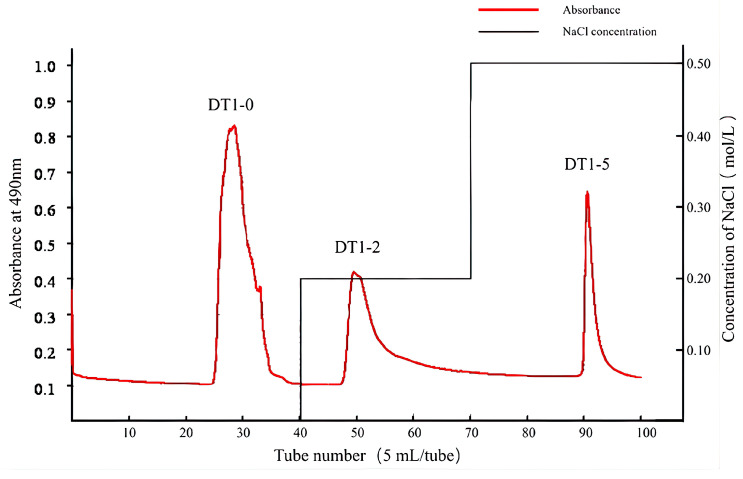
The chromatograms of crude EPS produced by *B. halotolerans* DT1, eluted using 0 M, 0.2 M, and 0.5 M NaCl solutions, on a DEAE-52 cellulose column.

**Figure 3:**
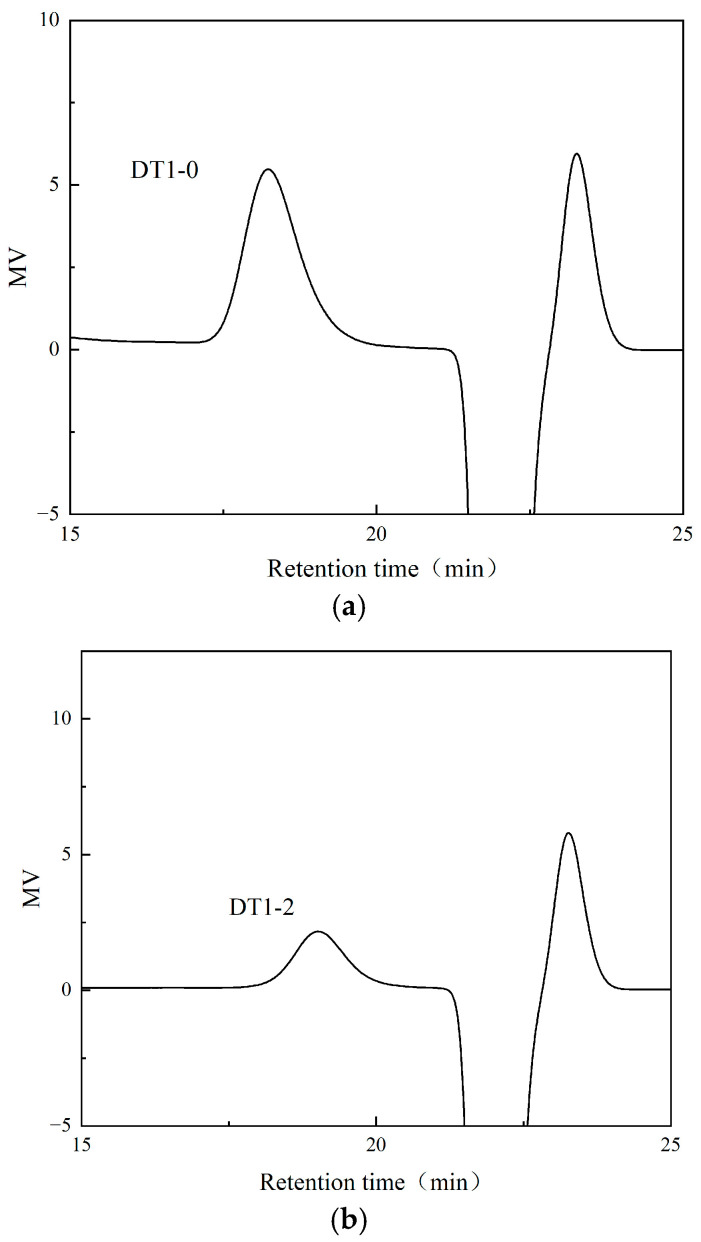

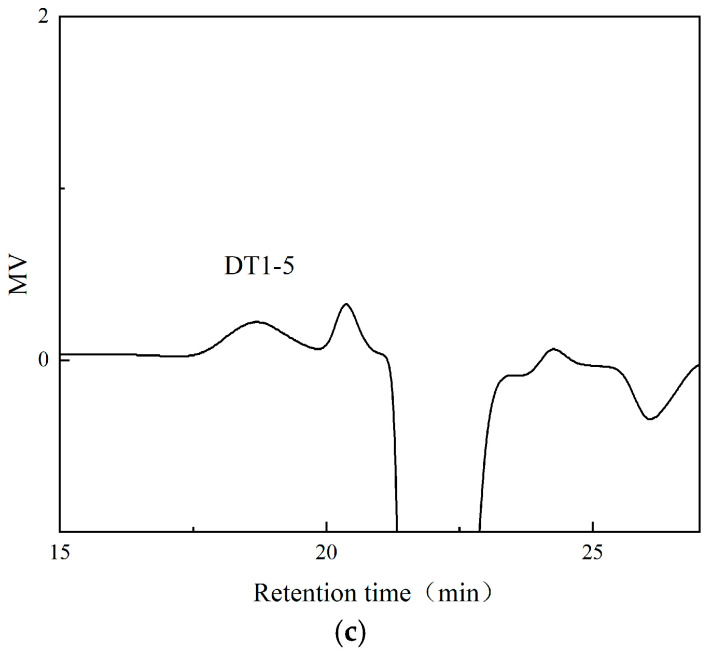
GPC spectra of three purified EPS through Sephadex G-25 column chromatography: (**a**) DT1-0, (**b**) DT1-2, and (**c**) DT1-5.

The EPS produced by *B. halotolerans* DT-1 was subjected to ethanol precipitation, Sevage protein removal, and dialysis. Subsequently, the separation was performed using a DEAE-52 cellulose column with elution using 0 M, 0.2 M, and 0.5 M NaCl solutions; three distinct polysaccharide fractions were isolated, namely, DT1-0, DT1-2, and DT1-5 (Figure 2). At the same time, no components were collected in the blank control group. Following a 2-day dialysis step, further purification was achieved through Sephadex G-25 gel filtration columns, resulting in relatively symmetric and singular peaks (Figure 3a–c). This indicates their homogeneity as individual polysaccharides. 

### 3.3. Analysis of EPS Molecular Weight (Mw) and Monosaccharide Composition and Glycosidic Linkage

**Figure 4:**
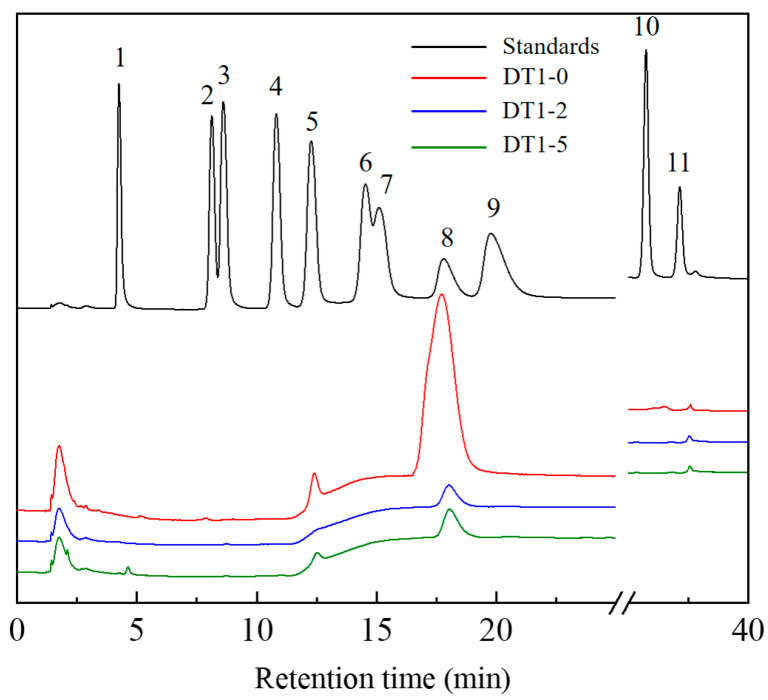
HPAEC-PAD chromatogram of hydrolyzed products of EPS produced by B. halophilic DT-1 and standard monosaccharides (1—fucose, 2—rhamnose, 3—arabinose, 4—galactose, 5—glucose, 6—xylose, 7—mannose, 8—fructose, 9—ribose, 10—galacturonic acid, 11—glucuronic acid).

**Figure 5:**
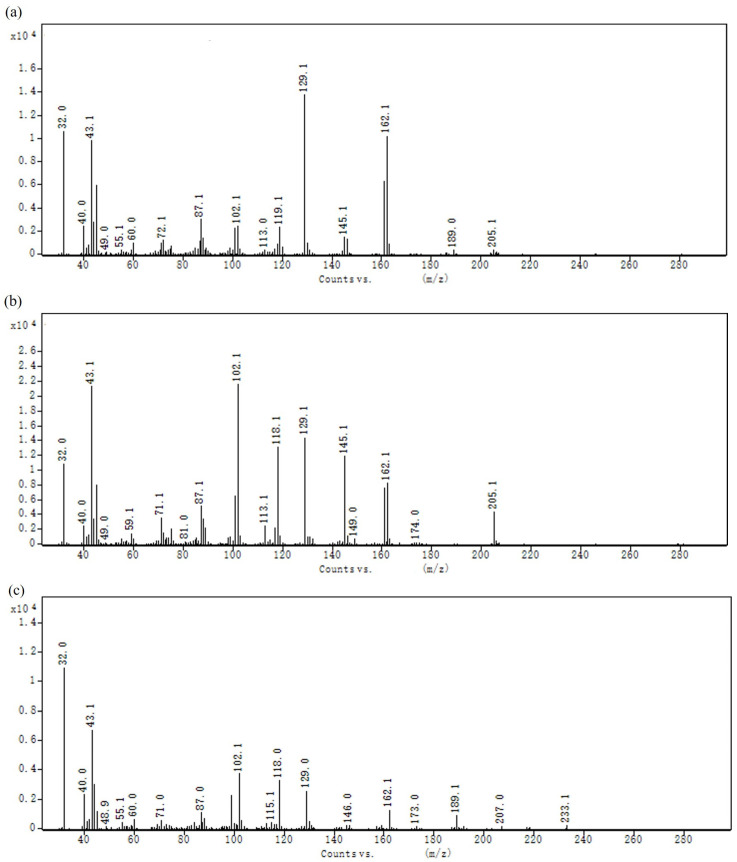

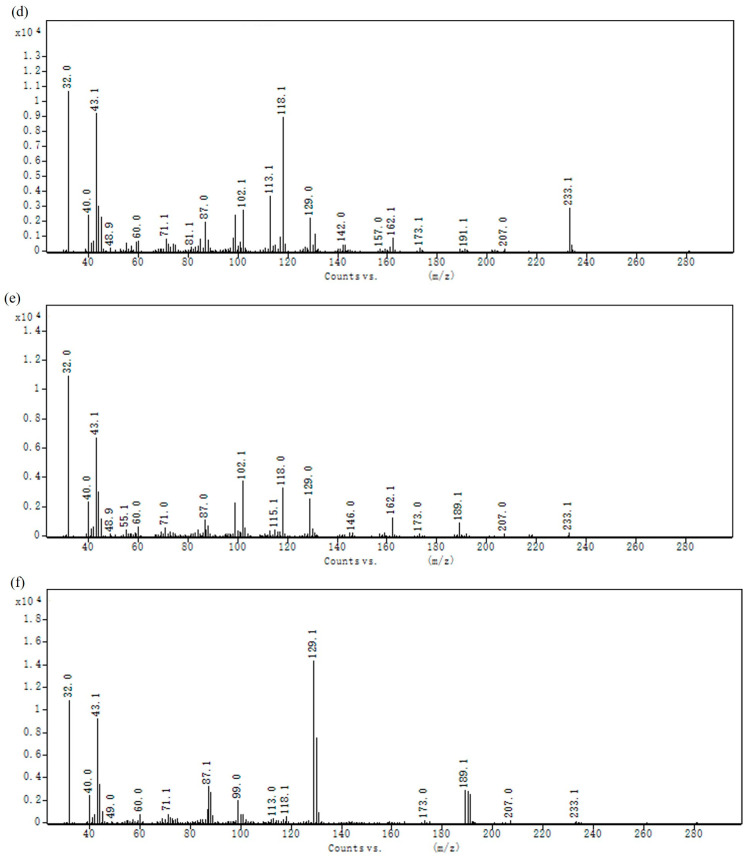
The mass spectrum of (**a**) 2,5-di-O-acetyl-1,3,4,6-tetra-O-methyl mannitol; (**b**) 1,5-di-O-acetyl-2,3,4,6-tetra-O-methyl glucitol; (**c**) 2,5,6-tri-O-acetyl-1,3,4-tri-O-methyl mannitol; (**d**) 1,4,5-tri-O-acetyl-2,3,6-tri-O-methyl glucitol; (**e**) 1,5,6-tri-O-acetyl-2,3,4-tri-O-methyl glucitol; and (**f**) 1,2,5,6-tetra-O-acetyl-3,4-di-O-methyl-hexitol (mannitol, glucitol).

The relevant molecular parameters of the three EPS are presented in Table 1, indicating that they all possess low molecular weights (*Mw*). Following acid hydrolysis, EPS were analyzed using the ICS 5000+ chromatographic system and compared to standard compounds (fucose, rhamnose, arabinose, galactose, glucose, xylose, mannose, fructose, ribose, galacturonic acid, glucuronic acid, and galacturonic acid lactone, at a concentration of 10 mg/mL). The results are illustrated in Figure 4. DT1-0 consists of fructose and glucose, with a molar ratio of approximately 93.40:6.60; DT1-2 is a type of fructooligosaccharide (FOS), composed entirely of fructose; and DT1-5 comprises a combination of fructose and glucose, with a molar ratio of approximately 98.60:1.40. The diversity of monosaccharide constituents suggests that the EPS produced by DT1 is a potential biologically active heteropolysaccharide. The methylation analysis of DT1-0, as shown in Table 2 and Figure 5, clearly indicates that →6)-β-D-Fruf-(2→ is the predominant building block of DT1-0. Additionally, trace amounts of β-D-Glcp-(1→, →4)-β-D-Glcp-(1→, and →6)-β-D-Glcp-(1→ were detected, consistent with the results of monosaccharide composition analysis [10].

### 3.4. Fourier-Transform Infrared (FTIR) Analysis

**Figure 6:**
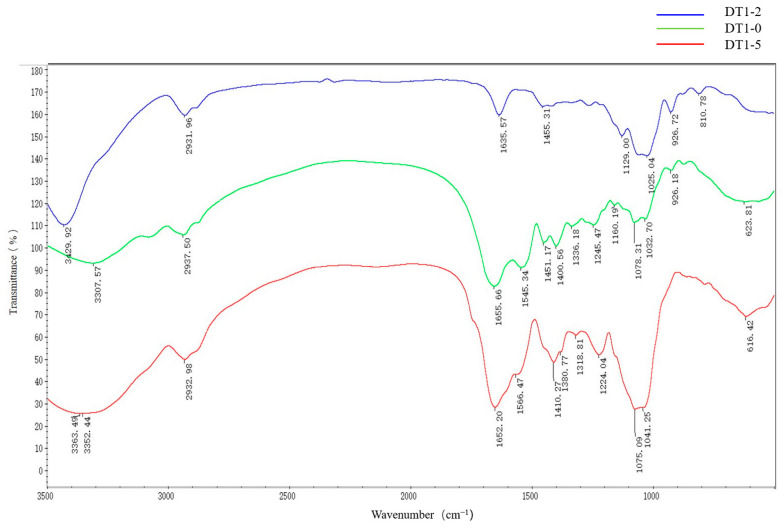
Fourier transform infrared spectroscopy (FT-IR) of EPS produced by *B. halotolerans* DT1: EPS-DT1-0, EPS-DT1-2, and EPS-DT1-5.

The FT-IR analysis is a fundamental method used for assessing the functional groups and compositional structures of EPS. Infrared spectroscopy was performed on three types of EPS as shown in Figure 6. The results indicated that these three EPS are distinct polysaccharides. In the spectrum of DT1-0, the strong band at 3429 cm^−1^ corresponds to the stretching vibration of hydroxyl groups (O-H) in EPS; the absorption peak at 2932 cm^−1^ indicates the stretching vibration of C-H bonds; the absorption peak at 1635 cm^−1^ is attributed to the stretching vibration of O-H bonds in crystalline water. The peak at 1455 cm^−1^ can be attributed to the stretching vibration of C=O, characteristic of carboxyl groups. Additionally, absorption peaks with the range of 1025–1130 cm^−1^ are related to the overlapping vibrations of C-O-C and C-O-H bonds, representing ring vibrations specific to polysaccharides, and the absorption peak at 810 cm^−1^ confirms the β-configuration of DT1-0 [11,12]. The DT1-2 and DT1-5 spectra exhibited similar trends, with noteworthy differences including the absorption peak between 1300 and 1500 cm^−1^ attributed to the stretching vibration of C=O, which is characteristic of carboxyl groups.

### 3.5. Nuclear Magnetic Resonance (NMR) Analysis

**Figure 7:**
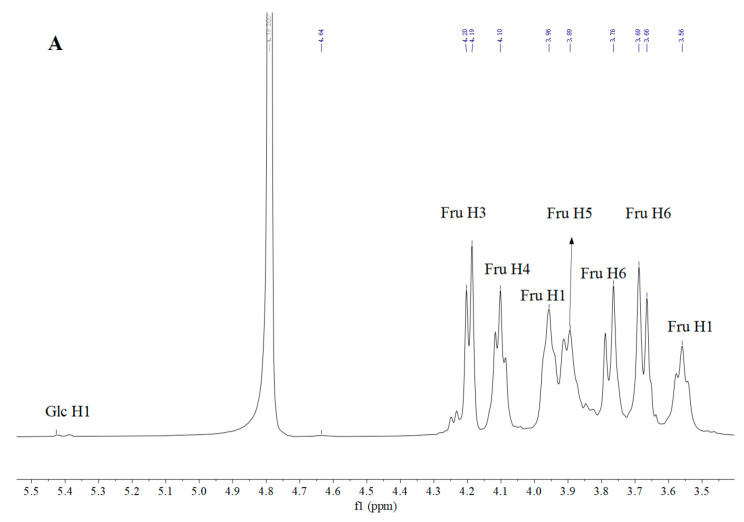

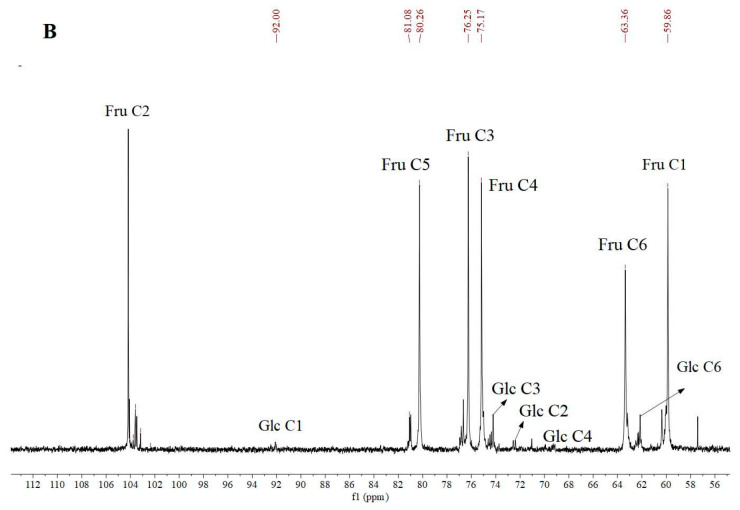
1D-1H (**A**) and 13C (**B**) nuclear magnetic resonance spectra of the major component EPS-DT1-0 produced by *B. halotolerans* DT1.

**Figure 8:**
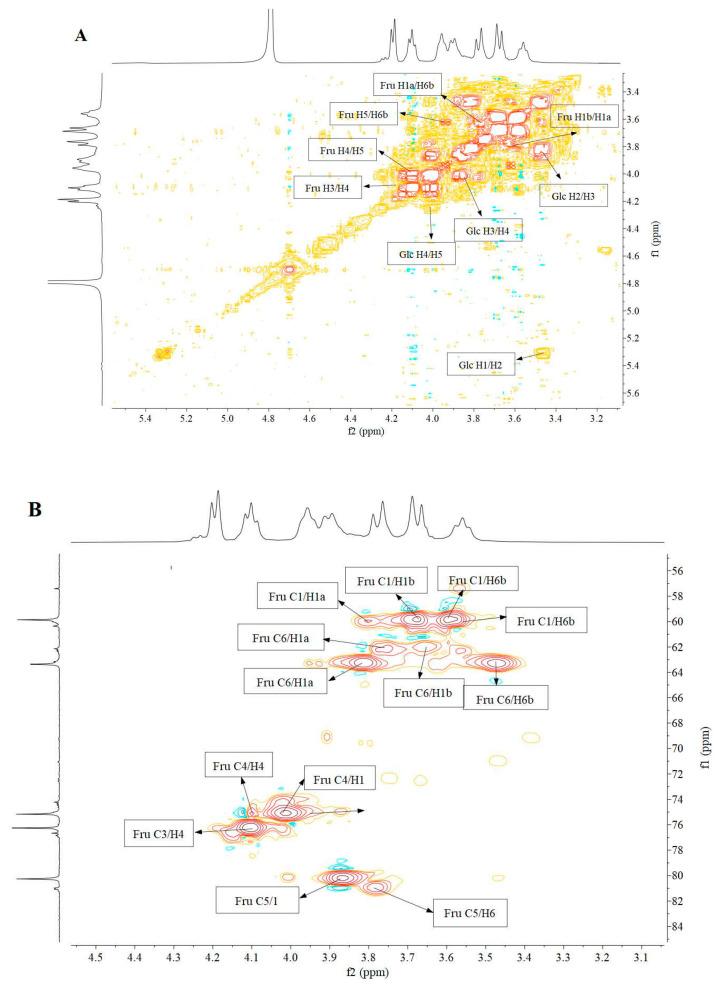

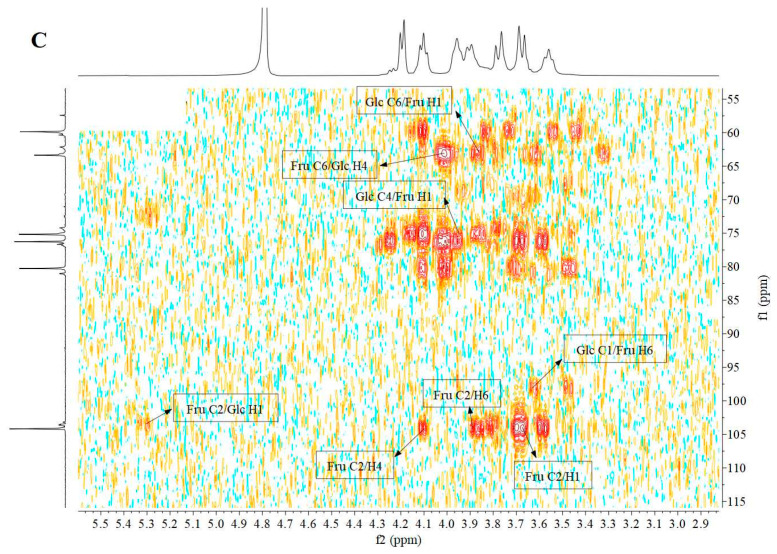
2D NMR spectra, (**A**) 2D-COSY, (**B**) HSQC, and (**C**) HMBC, of the major component EPS-DT1-0 produced by *B. halotolerans* DT1.

NMR analysis serves as an effective and reliable tool to elucidate detailed structural information of EPS, including the number of sugar residues, monosaccharide composition, anomeric configurations, and linkages. To further elucidate the structure of DT1-0, the predominant component of DT1, a series of NMR experiments were conducted, including 1D-1H, 1D-13C, 2D-HSQC, 2D-HMBC, 2D-NOESY, and 2D-COSY experiments, as illustrated in Figure 7 and Figure 8. The main signals in the 1H NMR spectrum were observed within the narrow range of 3.55–4.25 ppm, displaying seven proton signals (Figure 7A). No active hydrogen protons were detected in the anomeric proton region, indicating the characteristic features of fructooligosaccharides. The 13C NMR spectrum exhibited resonances of anomeric carbons between 103 and 105 ppm, confirming their β-type configuration, and the resonance at 104.15 ppm corresponded to the C-2 carbon of β-D-fructose, with several overlapping peaks attributable to the diverse positions of the same monosaccharide in the polysaccharide chain.

**Figure 9:**
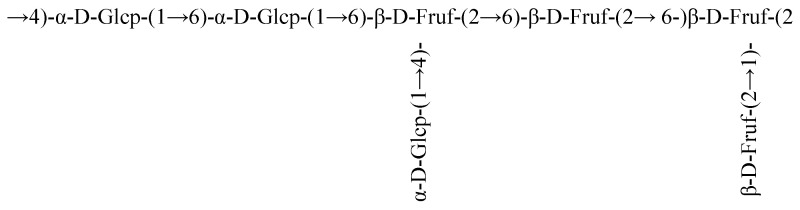
Predicted structure of the major polysaccharide component EPS-DT1-0 produced by *B. halotolerans* DT1.

Carbon signals of the sugar ring connected to oxygen (O) appeared in the range of 59.80–80.34 ppm, corresponding mainly to C-1, C-2, C-3, C-4, C-5, and C-6 positions near 59.86, 104.18, 76.25, 75.17, 80.26, and 63.36 ppm, respectively. According to the literature, the major signals near 59.86, 104.18, 76.25, 75.17, 80.26, and 63.36 ppm were attributed to C-1, C-2, C-3, C-4, C-5, and C-6, respectively. The distinctive signals at lower magnetic fields for C-6, C-2, and C-4, combined with the relative distances between carbon atoms, resemble the pattern of Levan fructooligosaccharides rather than Inulin type, indicating that DT1-0 is a Levan fructooligosaccharide. The results of COSY analysis (Figure 8A) indicated signals at 3.67/3.57 ppm, 4.11/3.99 ppm, and 4.01/3.86 ppm, corresponding to H-1(a,b)/H-6(b), H-4/H-5, and H-5/H-6(a) protons, respectively. The HSQC results depicted in Figure 8B demonstrated that the signal peaks at 3.67/59.76 ppm, 3.60/59.76 ppm, 3.82/63.31 ppm, 3.47/63.31 ppm, 4.02/75.09 ppm, and 3.87/80.25 ppm were assigned to H-1(a,b)/C-1, H-6(a,b)/C-6, H-4/C-4, and H-5/C-5, respectively. Additionally, the signal peaks at 3.76/62.18 ppm and 3.79/80.89 ppm confirmed the presence of the →4)-α-Glcp-(1→ and →6)-α-Glcp-(1→ residues. The HMBC results presented in Figure 8C revealed that the signal peaks at 4.10/59.96 ppm and 3.93/59.80 ppm were attributed to the correlations of H-4/C-1 and H-6/C-1, respectively, confirming the presence of the →1,6)-β-Fruf-(2→ residue. Moreover, the signal peak at 3.70/76.07 ppm was attributed to the correlation of H-1/C-4, confirming the existence of the →4)-α-Glcp-(1→ residue, and the signal peaks at 4.10/103.98 ppm, 3.67/103.98 ppm, and 3.57/104.98 ppm were ascribed to the correlations of H-4/C-2, H-1/C-2, and H-6/C-2, respectively, validating the presence of the Fruf-(2→ and →6-)-β-Fruf-(2→ residues. By employing the same methodology and comparing it with previously published NMR data [13,14,15,16,17,18,19], the chemical shifts of other residues’ protons and carbons were sequentially deduced, as detailed in Table 3. Based on these findings, a plausible structure for DT1-0 is proposed in Figure 9. The EPS-B108 produced by *Bacillus* SCU-E108 is composed of fructose, with a main chain consisting of β-(2→6)-linked fructose repeating units and a fructose side chain connected to C-1 through a β-(2→6) linkage, and the EPS-JN4 produced by *Bacillus* starch-degrading contains a mixture of fructose and glucose. Its main chain is composed of β-(2→6)-linked fructose residues, with a β-(2→1)-linked fructose side chain attached to every six residues. Numerous studies have investigated the production of fructooligosaccharides, yet a structure akin to that of DT1-0 (comprising a main chain of [4)-α-D-Glcp-(1→6)-α-D-Glcp-(1→6)-β-D-Fruf-(2→6)-β-D-Fruf-(2→6-)β-D-Fruf-(2→]), and the EPS with fructose and glucose linked to the third fructose and first glucose, respectively, via β-(2→1) and α-(1→4) glycosidic bonds, as observed in DT1-0, has not been reported.

### 3.6. Thermal Stability Analysis

**Figure 10:**
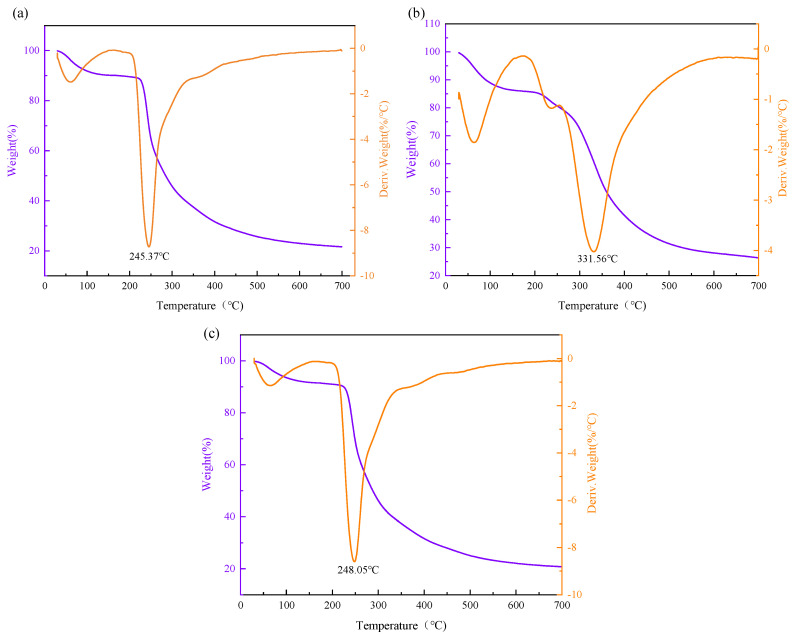
TGA of EPS (**a**) DT1-0, (**b**) DT1-2, (**c**) DT1-5 produced by *B. halotolerans* DT1.

The thermal stability of the EPS was assessed using thermogravimetric analysis (TGA), and their thermal analysis curves are depicted in Figure 10. 

The TGA curves exhibited a two-step degradation pattern for the three polysaccharides. For DT1-0, a slight initial mass loss of approximately 10% occurred between 50 and 100 °C, possibly due to the evaporation of residual moisture. No significant weight loss was observed between 100.86 and 245.37 °C, indicating the stability of DT1-0 below 245 °C. A rapid and substantial weight loss of approximately 58.5% occurred between 245.37 and 323 °C, indicative of the severe decomposition of DT1-0, potentially related to the cleavage of C-chain and hydrogen bonds. Similar to DT1-0, DT1-5 displayed thermal behavior with a higher stability threshold, remaining stable below 248.05 °C. Notably, DT1-2 exhibited remarkable thermal stability, as indicated by its rapid and substantial weight loss occurring only after 331.56 °C. All three EPS exhibited better thermal stability than EPS-B108, Levan S81, and GSBa-1, suggesting their potential utility in high-temperature processing within the food industry.

### 3.7. Scanning Electron Microscopy (SEM) 

**Figure 11:**
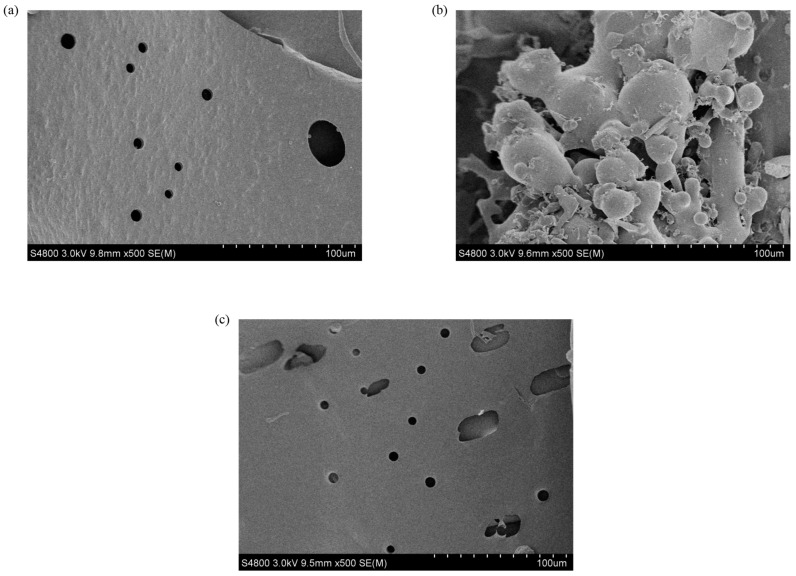
SEM analysis of EPS (**a**) DT1-0, (**b**) DT1-2, and (**c**) DT1-5 (500×).

The surface morphologies of EPS were unveiled using the SEM technique (Figure 11). For DT1-0, a layered structure with a smooth and porous surface is observed. DT1-2 exhibits an irregular vesicular structure with a relatively smooth surface, and DT1-5 displays a smooth surface with a regular laminar structure and porous features, though the pore size is larger than the surface of DT1-0. It is speculated that the reason why the apparent image of DT1-2 is so different from the other two polysaccharides is that the aggregation is caused by the increase in NaCl concentration during separation and purification. The porous nature of the structures suggests that EPS may possess favorable water-holding and oil-holding capacities, which had been investigated in subsequent experiments.

### 3.8. Atomic Force Microscopy Imaging

**Figure 12:**
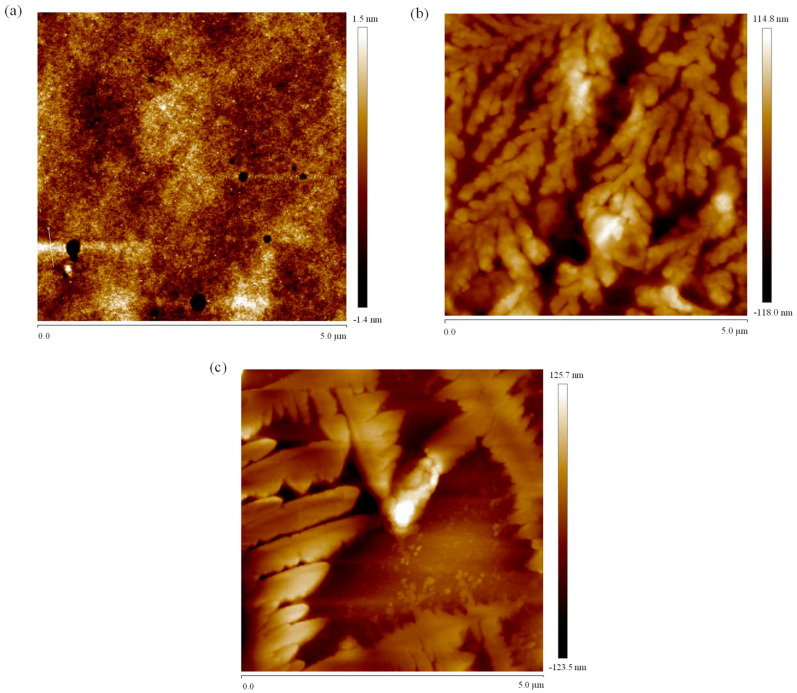
Two-dimensional AFM analysis of EPS (**a**) DT1-0, (**b**) DT1-2, and (**c**) DT1-5.

**Figure 13:**
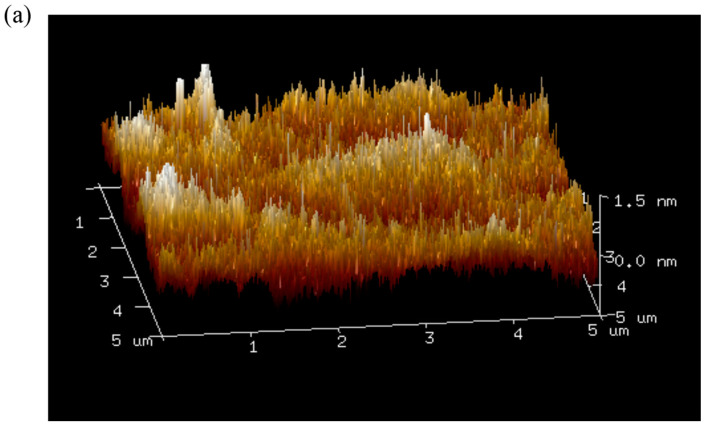

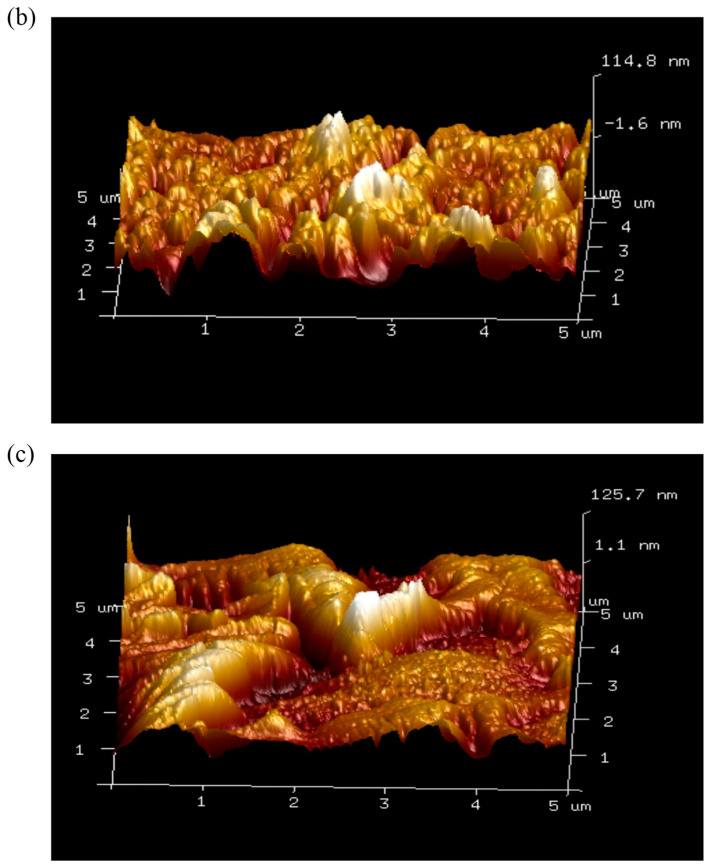
Three-dimensional AFM analysis of EPS (**a**) DT1-0, (**b**) DT1-2, and (**c**) DT1-5.

Atomic force microscopy (AFM) is a versatile tool that not only provides two-dimensional images but also enables the direct observation of three-dimensional surface profiles of polysaccharides under natural conditions. The AFM results of the EPS are illustrated in Figure 12 and Figure 13. As evident from Figure 13a, the surface structure of DT1-0 is compact and relatively flat, consistent with SEM images. Notably, as observed from Figure 12b,c, both DT1-2 and DT1-5 exhibit varying degrees of aggregation. This phenomenon could be attributed to the hydroxyl groups on the polysaccharide chains enhancing intermolecular and intramolecular interactions, potentially resulting in strong molecular aggregation. Since the two polysaccharides were purified using different NaCl concentrations, the presence of salt ions likely contributed to their curling aggregation. The addition of NaCl may bring some positive and negative charges, causing charge imbalance, resulting in the imbalance of hydroxyl charge and enhancing the interaction between molecules and molecules. The phenomenon might also be influenced by carboxyl groups, hydrogen bonding, and van der Waals forces, with the volume of aggregation reflecting the strength of molecular clustering. Additionally, polysaccharide molecules inherently tend to form stacking and aggregated structures for stability, and this tendency holds true for both DT1-2 and DT1-5.

### 3.9. Water Solubility Index (WSI) 

The solubility of EPS in water is primarily determined by its biological source, molecular structure, and *Mw*. All DT1-0, DT1-2, and DT1-5 exhibit high water solubility, estimated at 95.4 ± 1.5%, 92.8 ± 0.9%, and 97.1 ± 1.0%, respectively. These values exceed those of EPS003 [20], EPS produced by *Weissella cibaria* YB-1 [21], and EPS-B108. Moreover, highly water-soluble EPS typically exhibit strong hydrophilicity, which makes them well-suited for use in food applications as biologically active surfactants and stabilizers.

### 3.10. Water-Holding Capacity Evaluation

Water-holding capacity serves as a pivotal parameter to describe the physicochemical behavior of polysaccharides in aqueous solutions, encompassing the overall sum of water binding, fluid dynamic water retention, and physical entrapment. The water-holding capacity of DT1-0, DT1-2, and DT1-5 is estimated to be 504 ± 5.3%, 88 ± 7.6%, and 307 ± 10.6%, respectively. Both DT1-0 and DT1-5 exhibit elevated water-holding capacity, which is attributed to their porous structure and intermolecular hydrogen bonding interactions. Notably, compared to many polysaccharides such as EPS003, EPS-B108, YB-2 [22], and YB-1, DT1-0 demonstrated higher water-holding capacity. The high water-holding capacity of both DT1-0 and DT1-5 suggests promising applications as moisture retainers and stabilizers within the food industry. Conversely, DT1-2’s lower water-holding capacity can be attributed to its lower polymerization degree and absence of porous structure, indicating its potential to enhance the crispness of expanded products and minimize fracture susceptibility.

### 3.11. Lipophilicity Assessment

EPS with high lipophilicity are recognized for conferring attributes of smoothness, juiciness, and palatability, which are particularly desirable in various industrial applications. Specifically, the coconut oil-holding capacities were determined as 387 ± 1.9% for DT1-0, 306 ± 8.8%, and the olive oil-holding capacities were found to be 367 ± 2.6%, 318 ± 4.9%, and 440 ± 8.2% for DT1-0, DT1-2, and DT1-5, respectively. The underlying factors contributing to high lipophilicity encompass various aspects such as the permeability of EPS, the porous surface morphology of polysaccharides, and their chemical composition. EPS characterized by elevated lipophilicity serve as valuable additives that facilitate flavor retention, enhance the palatability of food products, and effectively absorb lipophilic components present in materials.

## 4. Conclusions

A strain (DT1) capable of producing EPS was isolated from preserved dried cabbages of Tianjin, which was classified as *B. halotolerans*, displaying optimal growth at 8% NaCl concentration and maintaining good growth even at 12% NaCl. Three distinct polysaccharide fractions, namely, DT1-0, DT1-2, and DT1-5, with *Mw* of 4.253 × 10^3^ Da, 1.722 × 10^3^ Da, and 2.582 × 10^3^ Da, respectively, were isolated and purified by DEAE-52 cellulose column eluted with solutions containing NaCl at concentrations 0 M, 0.2 M, and 0.5 M followed by purification on a Sephadex G-25 column eluted with distilled water. Structural analysis revealed that the predominant monosaccharide component in all three polysaccharides was fructose.

Among them, DT1-0, as the main component, is a heteropolysaccharide with a branched chain structure composed of fructose and glucose. Its surface exhibited a dense, smooth, and porous morphology, displaying superior thermal stability, water-holding capacity, and oil-holding capacity compared to many other EPS. The exceptional water-holding capacity of EPS Br42, reaching 510% [23], can be attributed to its porous microstructure, glycosidic bonds, and high polymerization degree. Therefore, it can be inferred that the heightened polymerization degree of DT1-0 may contribute to its superior water-holding capacity. DT1-2 was identified as a fructan with a smooth surface, characterized by irregular vesicular structures. AFM images revealed some degree of surface aggregation, and it exhibited remarkably high thermal stability (331.56 °C), moderate oil-holding capacity, and relatively low water-holding capacity. DT1-5 was composed of fructose and glucose residues and exhibited a similar aggregative surface, along with a porous structure featuring larger pores compared to DT1-0. It displayed notable thermal stability, water-holding capacity, and oil-holding capacity. Afshan Malick found that the EPS composition of Bacillus varies with different growth conditions and strains, resulting in differences in monosaccharide composition and *Mw* [24]. The fermentation products of *B. licheniformis* 14580 and *B. amyloliquefaciens* 23350 were completely different (glucan, galactose, and fructose) due to different fermentation environments (different ratios of medium). Xiaoqi Zhao discovered that the EPS produced by B. halotolerans LR-1 is a heteropolysaccharide primarily composed of mannose (13.58%) and glucose (82.95%) [6]. It is speculated that the proportion of fructose produced by *Bacillus* in the high salt environment is higher.

The favorable high water-holding capacity, superior thermal stability, and substantial oil-holding capacity underscore the potential for utilization in food processing industries. Additionally, the low water-holding capacity contributes to enhancing the crispness of expanded products while minimizing the likelihood of rupture. Water-holding capacity and OHC suggest some possibilities about the use of EPS as ingredients in food products, e.g., dietary EPS with high OHC allow the stabilization of high-fat food products and emulsions. Dietary EPS with high WHC can be used as functional ingredients to avoid synaeresis and modify the viscosity and texture of some formulated food [25]. These findings provide further insights into DT1 and its EPS products, thereby expanding possibilities for applications in the fields of food processing and the chemical industry.

## Figures and Tables

**Table 1:** The relevant molecular parameters of the three EPS produced by *B. halotolerans* DT1: weight-average molecular weight (*Mw*), number-average molecular weight (*Mn*), Z-average molecular weight (*Mz*), and peak molecular weight (*Mp*).

Types of Polysaccharides	*Mw*	*Mn*	*Mz*	*Mp*
DT1-0	4253	3996	4098	3876
DT1-2	1722	1326	2164	1636
DT1-5	2582	2069	3181	2416

**Table 2:** The results of sugar residue analysis of EPS-DT1.

Residue	(PMAA)	*Mw*	Peak Area	Peak Height	Peak Width	Relative Hazard Molecular Weight	Relative Molar Ratio (%)
Fruf-(2→	2,5-di-O-acetyl-1,3,4,6-tetra-O-methyl mannitol	323	1,516,418.11	457,566.68	0.383	4694.793	7.815
		323					
Glcp-(1→	1,5-di-O-acetyl-2,3,4,6-tetra-O-methyl glucitol	323	2,132,435.17	611,101.17	0.104	6601.966	10.990
→6)-Fruf-(2→	2,5,6-tri-O-acetyl-1,3,4-tri-O-methyl mannitol	351	15,878,216.23	4,374,779.81	0.376	45,237.083	75.304
		351					
→4)-Glcp-(1→	1,4,5-tri-O-acetyl-2,3,6-tri-O-methyl glucitol	351	289,025	77,234.48	0.150	823.433	1.371
→6)-Glcp-(1→	1,5,6-tri-O-acetyl-2,3,4-tri-O-methyl gluctiol	351	189,399.83	49,851.83	0.181	539.601	0.898
→1,6)-Fruf-(2→	1,2,5,6-tetra-O-acetyl-3,4-di-O-methyl-hexitol (mannitol, glucitol)	379	824,629.15	136,153.77	0.239	2175.803	3.622

**Table 3:** 1H and 13C chemical shifts, glycosidic linkage types, and derivative analysis of the major components of EPS-DT1 produced by *B. halotolerans* DT1.

Residue	δ^13^C/^1^H (ppm)					
	1	2	3	4	5	6
β-D-Fruf-(2→	59.96 3.77, 3.67	104.08	76.71 4.20	74.90 4.08	80.91 3.96	63.18 3.89, 3.55
α-D-Glcp-(1→	92.12 5.42	74.21	72.37 4.23	71.04 4.09	61.21 3.94	57.42 3.90
→6)-β-D-Fruf-(2→	59.81 3.76, 3.69	104.18	76.24 4.18	75.11 4.10	80.25 3.96	63.33 3.89, 3.56
→4)-α-D-Glcp-(1→	92.53 5.38	74.35	72.58 4.23	70.96 4.11	61.15 3.94	57.27 3.90
→6)-α-D-Glcp-(1→	92.27 5.26	74.47	72.55 4.25	71.16 4.09	61.36 3.94	57.48 3.90
→1,6)-β-D-Fruf-(2→	60.17 3.79, 3.67	103.59	76.97 4.20	75.37 4.11	81.17 3.96	63.50 3.89, 3.54

## Data Availability

Data are contained within the article.
